# How Can I Help You? The Influence of Situation and Hostile Sexism on Perception of Appropriate Gender of Conversational Agents

**DOI:** 10.5334/irsp.669

**Published:** 2023-07-12

**Authors:** Mathieu Pinelli, Elisa Sarda, Clémentine Bry

**Affiliations:** 1 Université Grenoble Alpes, Université Savoie Mont Blanc, CNRS, LPNC, 38000 Grenoble, France; 2 Nantes Université, Université Angers, Laboratoire de psychologie des Pays de la Loire, LPPL, UR 4638, F–44000 Nantes, France; 3 Université Savoie Mont Blanc, Université Grenoble Alpes, LIP/PC2S, 38000 Grenoble, France

**Keywords:** Ambivalent Sexism, Gender Biases, Conversational Agents

## Abstract

Conversational agents (CAs) are increasingly being developed on commercial websites nowadays. We tested in two studies whether gender stereotypes apply to non-gendered CAs. In the first study, participants evaluated whether CAs are expected to display more masculine or feminine characteristics in situations designed to be stereotypically male or female. The sexist attitudes of the respondents were also measured. As predicted, participants perceived that a CA should be more masculine in stereotypically male situations and more feminine in stereotypically female situations. Moreover, we found that hostile sexism but not benevolent sexism moderated the effect of the gendered situation. The second study replicated the results while addressing the limits of Study 1, showing the robustness of these effects. These findings are consistent with models of gender stereotypes in humans and robots and show for the first time a moderation effect of (hostile) sexism in a customer service context with CAs. The processes involved in human relationships seem relevant in a digital environment that involves CAs. Researchers and professionals should work together to avoid reproducing and perpetuating gender stereotypes when developing CAs.

## Introduction

Interactions between machines and humans have aroused many fantasies since the early development of computers, robots, and artificial intelligence. The claims that ‘Machines will replace humans’ or ‘we will no longer differentiate between humans and machines’ are often heard in everyday talk. Fiction stories about machines taking over humans are numerous (e.g., *The Terminator, The Matrix*, and *Westworld*, to name just a few films and TV shows).

Robots and artificial intelligence applications are increasingly being used on-line to help users with customer services and to simulate a realistic human presence. We focus in this paper on conversational agents (CAs) designed to interact with humans using natural language (Dale, 2016; Feine et al., 2019). Conversational agents are almost a must-have on a commercial website these days (e.g., there were 300,000 CAs on Facebook in 2018),^1^ and they have positive consequences on users by increasing satisfaction and giving the feeling of a social presence (Chung et al., 2020; Feine et al., 2019). Conversational agents can be found in the form of personal assistants (e.g., Cortana, Alexa, Siri), as customer services support, in multiple technical support roles (smartphones, tablets, or computers), and in various fields, such as education, healthcare, and marketing (Bickmore & Gruber, 2010; Chung et al., 2020; Provoost et al., 2017; Tegos & Demetriadis, 2017).

Conversational agents are increasingly sophisticated and are used on a daily basis in direct contact with users in a B2C context (e.g., Chung et al., 2020). The development of CAs requires trade-offs between different technical and social features (Feine et al., 2019). One of the inevitable questions lies in relation to a possible gender for CAs, as CAs are used to increase the feeling of a human social presence and human interactions are coloured, for better or for worse, by gender and gendered behavioural expectations. Users may therefore expect gendered features for CAs; at least, developers seem to think so and have therefore produced gendered CAs.^2^ In this paper, we question whether people actually expect a gendered CA and the factors that would trigger such gendered expectations. No experimental study to our knowledge has studied the gendered expectations in relation to CAs before. The literature about gender features in human-human interactions and in robot-human interactions can help delineate what we can expect from CAs.

### Gender in Human Interactions

Gender and its associated beliefs are central in our social relationships (Eagly & Wood, 2016; Ellemers, 2018). Men and women are believed to be similar in some ways but very different in many other ways. These beliefs influence not only our perceptions, but also our behaviour (e.g., Ellemers, 2018; Spencer et al., 2016), thus reinforcing themselves as men and women adopt gendered social roles (Eagly & Wood, 2012). These gendered social roles give the impression that they are innate and inevitable, and therefore seem to be inherent in our society (Eagly & Wood, 2016).

Gender stereotypes are both descriptive (that is, what people are) and prescriptive (that is, what people should be; Prentice & Carranza, 2002; Eagly & Karau, 2002; Ellemers, 2018). Extensive research has identified two core dimensions in social perception: Communion and Agency (or warmth and competence; see, for instance, Abele et al., 2008; Fiske et al., 2007; Judd et al., 2005). Communion is related to warmth, sympathy, emotional sensitivity, and concern with others, whereas Agency is related to competence, assertiveness, confidence, and self-control (e.g., Cuddy et al., 2008; Eagly & Karau, 2002). Social perception research has found that men are described as more agentic than women and that women are described as more communal than men (Eagly & Steffen, 1984; Ellemers, 2018). Furthermore, matching the prescription, men’s behaviour is expected to be related to competence and agency, while women’s behaviour is expected to be related to warmth and care (Prentice & Carranza, 2002). These gender norms define what traits are acceptable (or unacceptable) for men and women, and breaking the gender norms can lead to prejudice (e.g., Eagly & Karau, 2002). Gender norms define the behaviour that women and men should display and, thus, the situations that conform to each gender. Situations involving care and communality are deemed more appropriate for women, and reciprocally women are perceived as better suited for care and warmth situations. On the other hand, situations that require competence, assertiveness, and confidence are deemed more appropriate for men, and reciprocally men are perceived as better suited for competence and assertiveness situations (Eagly & Wood, 2012; Ellemers, 2018).

From the gender stereotype literature, we can infer that some people could expect an interaction agent (here a CA) to match a specific gender social role. The gender role could be cued, for instance, by the situation at hand. A situation involving warmth and care would cue to a female gender role, while a situation involving competence and assertiveness would cue to a male gender role. Interestingly, CAs are used in a variety of situations, with some situations being more related to warmth and care (e.g., using the guarantee attached to a hairdressing appliance) and other situations being related to competence and assertiveness (e.g., financial services allowing customers to save and invest money). Users could expect the CA to conform to a female gender role in a warmth-related situation, whereas they may expect the CA to conform to a male gender role in a competence-related situation. At least, those predictions would hold if social roles were to be applied to artificial intelligence and machines. The literature on robot-human interaction may help us understand whether there is solid ground for such hypotheses.

### Gender in Human-Robot Interactions

Some studies have shown that people react to computers in the same way as they do to humans (Feine et al., 2019; Nass & Moon, 2000), and that people are able to interact with computers in the same way as they do with humans (Nass et al., 1997). The *Computers Are Social Actors* (CASA) model states that people interacting with computers have social reactions similar to human social interactions according to social cues like voice, gesture, physical design, or the apparent ‘gender’ (e.g., Eyssel & Hegel, 2012; Feine et al., 2019; Gong, 2008; Nass et al., 1997).

Voice is an important social cue defining personality and gender attribution. Nass et al. (1997) found that a high-pitched synthetic voice was associated with a ‘female’ computer, whereas a low-pitched synthetic voice was associated with a ‘male’ computer. Their study showed that humans react to a computer by applying the same social rules they usually reserve for social interactions between humans (see also: Nass & Moon, 2000). More recently, Eyssel and Hegel (2012) tested the effect of gendered facial features of robots on perception and description. They reported that short-haired robots (i.e., those with a male facial feature) were perceived as more agentic than long-haired robots (i.e., those with a female facial feature), which were perceived as more communal. Furthermore, tasks (such as repairing technical equipment) were perceived as more suitable for a ‘male’ robot and conversely female-dominated tasks dominated by women (such as household maintenance) were perceived as more suitable for a ‘female’ robot. More recently, Bernotat et al. (2021) showed how body shape also influences the perception of a robot. Their results indicated that stereotypically female activities and communal attributions were associated with a robot with a female body shape rather than with a male body shape. Furthermore, they showed that benevolent sexism (but not hostile sexism) marginally affected the agency attribution. Correlation analysis showed that the higher benevolent sexism was, the more agency was attributed to the robot.

Therefore, gender stereotypes are applied to robots. Several studies have extended this research to CAs, showing that social features affect users’ satisfaction, but also their perceptions of truthfulness, credibility, and social presence (Araujo, 2018; McDonnell & Baxter, 2019; Toader et al., 2020; Verhagen et al., 2014). Humans can interact with CAs in a natural language and adopt behaviours they usually have with their peers, that includes abuse, harassment, and mistreatment (Brahnam & De Angeli, 2012). Verbal abuse and sexual communication during interaction with CAs are common (De Angeli & Brahnam, 2008). For example, Brahnam and De Angeli (2012) showed that 18% of the conversation was focused on sexual attention and negative stereotypes with female CAs compared to 10% with male CAs and only 2% with non-gendered CAs.

Overall, the literature shows that people interact with CAs or robots in a similar way as they do with human fellows. Sometimes, these interactions with CAs or robots can also exacerbate negative social processes such as gender stereotypes, harassment, or gender-based division of labour with the consequence of reproducing and reinforcing sexism daily in our society (Brahnam & De Angeli, 2012; Eyssel & Hegel, 2012; Nomura & Suzuki, 2022).

It appears that gender roles are used to interact with CAs and that gender stereotypes are applied to CAs as well as humans. Human features (e.g., a voice and/or a face) are implemented to improve the user’s experience, giving a personalized service anytime and anywhere (e.g., Chung et al., 2020), and these human features can increase inferences of social roles. However, with CAs, the interactions are generally in a written form, through a chat, which means that such human features are not relevant. There might sometimes be an avatar displaying a male or female character, but this gendered avatar is not systematically present. Therefore, most CAs could be more gender neutral than robots. Unable to rely on gendered features, will people still project gender roles on CAs? When the CA has no gender feature, is the (gendered) situation enough to trigger gender expectations toward the conversational agent? Actually, we believe that adherence to sexism could play a role.

### Sexist Attitudes

Gender stereotypes have been extensively studied in human interactions, and some studies have extended that literature to robot interactions. In human interactions, the use of gender stereotypes depends on sexist attitudes. Sexism was once studied as a unitary dimension, but Glick and Fiske (1996) offered a more nuanced definition with their theory of ambivalent sexism. They proposed that two sorts of sexism coexist, as the two faces of the same coin: hostile sexism and benevolent sexism. Hostile sexism matches the more traditional sexist attitudes reviewed in the literature, comprising a negative attitude towards women, with feelings of antipathy and a fear that women will take power over men (Glick & Fiske, 1996). Hostile sexism can be expressed through discrimination in employment. Studies have shown, for example, that individuals higher in hostile sexism are less likely to recommend a female candidate for a managerial position (Masser & Abrams, 2004). Benevolent sexism, on the other hand, can be seen as a ‘more positive’ attitude toward women, associated with chivalry and paternalistic attitudes (Glick & Fiske, 1996). In this form of sexism, women are perceived as having a higher moral purity than men and as too fragile to undertake tasks involving strength (protective paternalism). They are also perceived as creatures without whom men cannot be complete and possess qualities that men do not possess. Those individuals higher in benevolent sexism therefore assign women to less challenging tasks (King et al., 2012), and perceive men as more agentic and women as more communal (Rudman & Kilianski, 2000). Benevolent sexism can be seen as more positive than hostile sexism, though both attitudes involve prejudice against women, placing them below men (e.g., Stermer & Burkley, 2015). For example, by describing women as warmer than men, benevolent sexism suggests that women are less competent than men (Kervyn et al., 2012).

## The Current Research

Gender stereotypes infuse our social life and influence our interactions in a variety of contexts, including marketing, workplaces, and robot interactions (Bernotat et al., 2021; Grau & Zotos, 2016; Koch et al., 2015). With digital growth, the question of the influence of gender stereotypes in digital contexts involving virtual CAs is of importance. Several previous studies have focused on gender stereotypes in robots (e.g., Eyssel & Hegel, 2012), but no study has experimentally tested gender biases and sexist attitudes with CAs. We believe that there is little reason to expect that gendered CAs would not trigger gender stereotyping. However, we wondered whether neutral CAs would still be the target of sexist stereotypes and if stereotyping would be predicted by the participants’ own level of sexist attitude (i.e., hostile and benevolent sexism). We reasoned that according to the commercial service one is looking for (e.g., advice about saving money vs. finding beauty products), people could consider the situation as stereotypically masculine or feminine. Our two studies aimed to test the impact of stereotypically male and female situations on the perception of appropriate features for CAs (gender, warmth, and competence) and the moderator effect of ambivalent sexism, represented by hostile and benevolent sexism.

In this paper, we extend previous work and test whether perceptions of gender-undefined CAs are also influenced by gender stereotypes and sexist attitudes. In two studies, participants were presented with several stereotypically ‘gendered’ situations in which they had to indicate the most appropriate characteristics (i.e., gender, warmth, and competence traits) for the CA. We formulate the following hypothesis:

H1a: Participants would consider the male gender to be more appropriate for the CA in stereotypically male situations and the female gender to be more appropriate in stereotypically female situations.

H1b: Participants would deem warmth features more appropriate for the CA in stereotypically female situations and competence features more appropriate in stereotypically male situations.

H1c: The effect of stereotypically male and female situations would be moderated by sexist attitudes such that the more sexist (hostile and/or benevolent) the participant, is the more they would rely on gender stereotypes in their evaluation of the appropriate characteristics of the CAs.

### Study 1

#### Method

##### Participants

A power analysis was performed using G*Power 3.1 (Faul et al., 2007) with a small to moderate effect size of *f^2^* = .10, using a within-subjects design and based on the literature on sexism (e.g., McCarty & Kelly, 2015). This power analysis suggested that we needed 114 participants for a power level of .80. Thus, 117 participants took part in our online study. French-speaking participants were recruited on the Prolific platform (only participants with 95% positive rates were included) and they received £0.84 for their participation. Fifteen participants were excluded after an initial sort,^3^ so the final sample included 102 participants (*M_age_* = 30.54, *SD* = 10.56; 38 women and 64 men). As we do not meet the number of participants recommended by the power analysis, we performed a sensitivity analysis to indicate what effect size was detectable with the final sample at 80% power (threshold of .05, 102 participants, and 20 predictors in the linear model) using G*Power. The analysis indicated that with this design, the minimum effect we could detect would be *f^2^* = .11.

#### Material and Procedure

To reduce participants’ suspicions towards the purpose and hypothesis of the study, the cover story presented the two parts as two separate studies, which were said to be combined for economic reasons. The alleged goal of the first ‘study’ was to validate questionnaires in different domains (marketing, ecology, gender perception). The participants were informed that they would randomly answer only one of three possible questionnaires. Actually, they always answered the gender perception questionnaire, which consisted of the Ambivalent Sexism Inventory (Glick & Fiske, 1996) validated in French (Dardenne et al., 2006). We used the short version of Rollero et al. (2014). The scale consists of two dimensions: hostile sexism and benevolent sexism. Both subscales are composed of six items (e.g., *women seek power by having control over men*; *many women have a kind of purity that men do not*). The participants provided a response for each item on a scale from 1 (not at all) to 6 (completely) and obtained a mean score for hostile sexism and a mean score for benevolent sexism.

The participants then moved on to the alleged Study 2, presented as a marketing research about the development of online CAs. A conversational agent was defined as ‘a computer program capable of conducting a conversation’, so that all participants had the same representation of a CA. The participants were told that they would be presented with different online situations in which a customer (of unspecified gender)^4^ would resort to a CA to answer their request. The participants’ task would be to indicate the CA’s most appropriate features to match the customer’s needs in each situation. Participants were instructed to answer from the customer’s point of view and not from their own, in order to limit social desirability bias (Fiske et al., 2002). Nine situations were presented in a random order to each participant (using a within-subjects design). The situations^5^ were related to online banking services and to retail websites, and were designed to conform to stereotypically male (*N* = 3), female (*N* = 3), or neutral (*N* = 3) gender norms.

For each situation, the participants answered a questionnaire on the CA’s appropriate features. They first evaluated the appropriate CA gender (from 1 = male to 5 = female), and its appropriate age (in its twenties, thirties, forties, or fifties). Then participants were required to rate the relevance of eight traits for the CA on a Likert scale ranging from 1 (not at all) to 5 (very much). Agency and communion traits were used to study gender stereotypes in robots. However, agency is related to actions in the world, which is not relevant to conversation agents. We therefore chose traits related to competence and warmth instead, as they are more general (see Cuddy et al., 2008). These items were adapted from Fiske et al. (2002). *Trustworthy, friendly, well-intentioned*, and *warm* evaluated the warmth dimension, and *competent, intelligent, capable*, and *efficient* evaluated the competence dimension.

Participants then completed a post-experimental questionnaire. We measured the attitude toward CAs with four items adapted from Venkatesh et al. (2003) on a 7-point Likert scale, and one item measured the frequency of use (from 1 = Never to 5 = Very often). The five items comprised an attitude index (α = .88). The higher the score, the more positive is the participant’s attitude towards CAs. We checked for suspicions regarding the actual/alleged goals of the study and the possible influence between the different parts, with three open questions. The answers were coded by the authors and rated from 0 = not suspicious to 3 = completely suspicious. Finally, a socio-demographic questionnaire collected the age, sex, socio-professional category, and nationality of each participant.

#### Results

##### Analysis Plan

Given our design, we used linear mixed-effects models with fixed and random effects variables. All analyses were conducted in R, using mixed-effects models with the lme4 package (Bates et al., 2021). Mixed models allow the use of fixed-effect variables (as in ANOVA) and random-effect variables.

##### Dependent Variables

We computed a perceived appropriate gender (1 = male to 5 = female), an appropriate warmth index (mean evaluation of warmth traits from 1 = not at all to 5 = very much), and an appropriate competence index (mean evaluation of competence traits from 1 = not at all to 5 = very much) for each scenario.

##### Independent Variable with Random Effects

The participants and the nine situations were variables with random effects. Therefore, we included in the model the estimation of their intercept and slope by situation or slope by sexism level, respectively.^6^

##### Independent Variable with Fixed Effects: The Situations

We created two contrasts to test a linear trend from stereotypically male to the stereotypically female situations through the neutral one. We coded the first contrast C1: female = +1; neutral = 0; male = –1, and the residual contrast C2: female = –1; neutral = +2; male = –1. If the trend is linear, we expect that C1 is significant and C2 to be not significant.

Both contrasts C1 and C2, participants’ gender (–1 = woman, +1 = man), benevolent sexism (centred), hostile sexism (centred), suspicion level (centred), and attitude toward CAs (centred) were entered as fixed effects in the linear mixed-effect model (see Judd et al., 2012).

All measures showed good internal consistency (see Table 1). Following Judd et al. (2012) and Judd et al. (2017), we compared models with and without each random parameter in order to retain the most conservative model. We followed the same rationale with fixed effects.^7^ The results corresponding to the tested hypotheses are presented in Table 1 (see mixed-effects models on our OSF page).

**Table 1:** Means (*SD*) and Cronbach’s alpha of variables included in the model (Study 1).


	MEAN (*SD*)	CRONBACH’S ALPHA

Hostile sexism	2.58 (1.21)	.90

Benevolent sexism	2.88 (1.11)	.82

Appropriate Competence	4.50 (0.50)	.73

Appropriate Warmth	4.07 (0.60)	.74

Attitudes toward CAs	4.52 (1.14)	.88


##### The Appropriate Gender of the Conversational Agent

**Figure 1:**
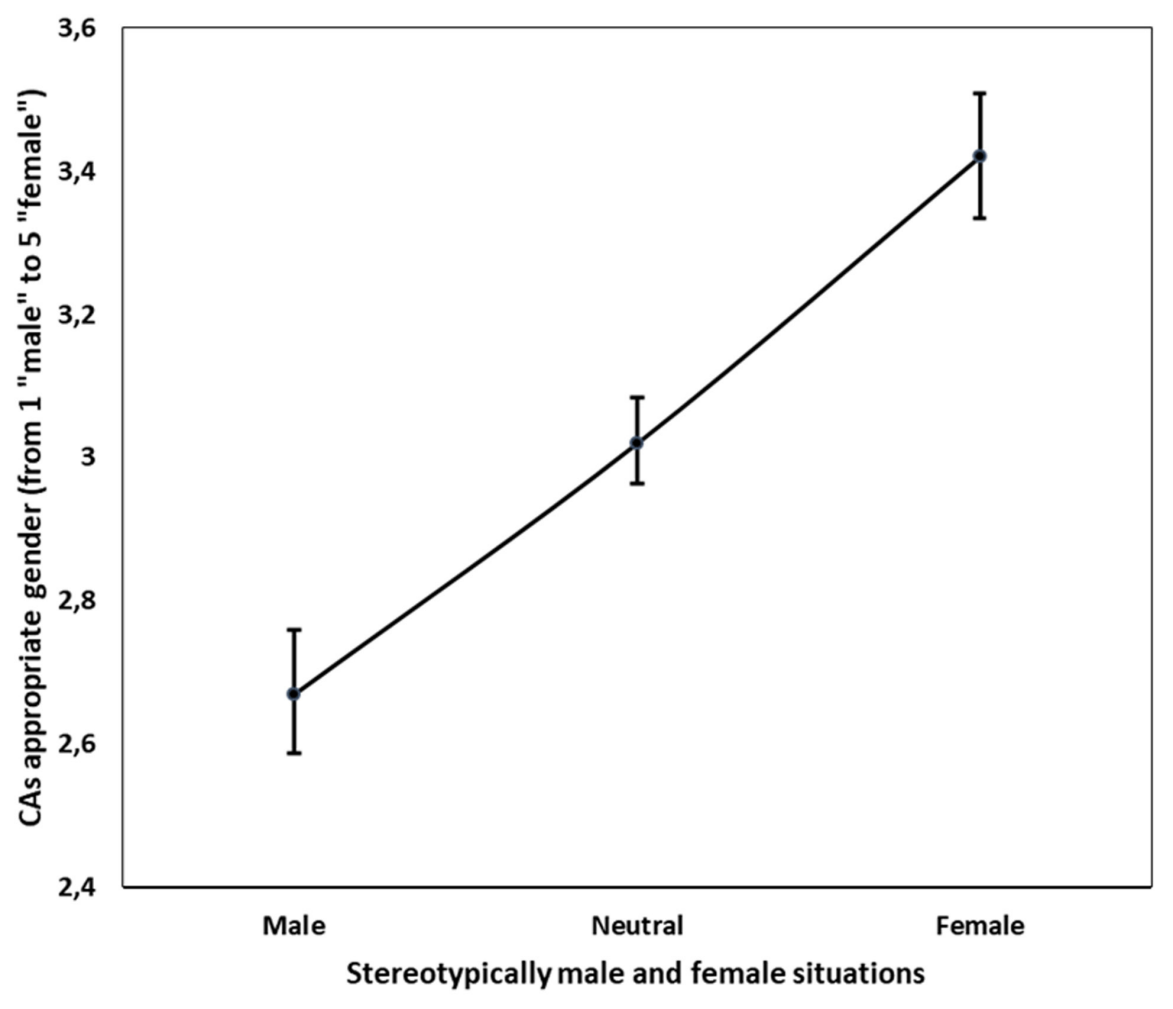
Effect of the stereotypical situations on the CA’s appropriate gender (bars represent confidence intervals).

Suspicion level, gender of participants, attitudes towards CAs, and benevolent sexism did not have a valuable input in the model and were therefore discarded. We found a significant effect of C1, *t* = 3.12, *p* = .016, but not of C2, *p* = .90. As expected, we found a significant effect of stereotypical situations. We observed that the appropriate gender linearly increases toward femininity (Figure 1) when passing from stereotypically masculine situations (*M* = 2.67; *SD* = 0.76) to stereotypically feminine situations (*M* = 3.42; *SD* = 0.78).

**Figure 2:**
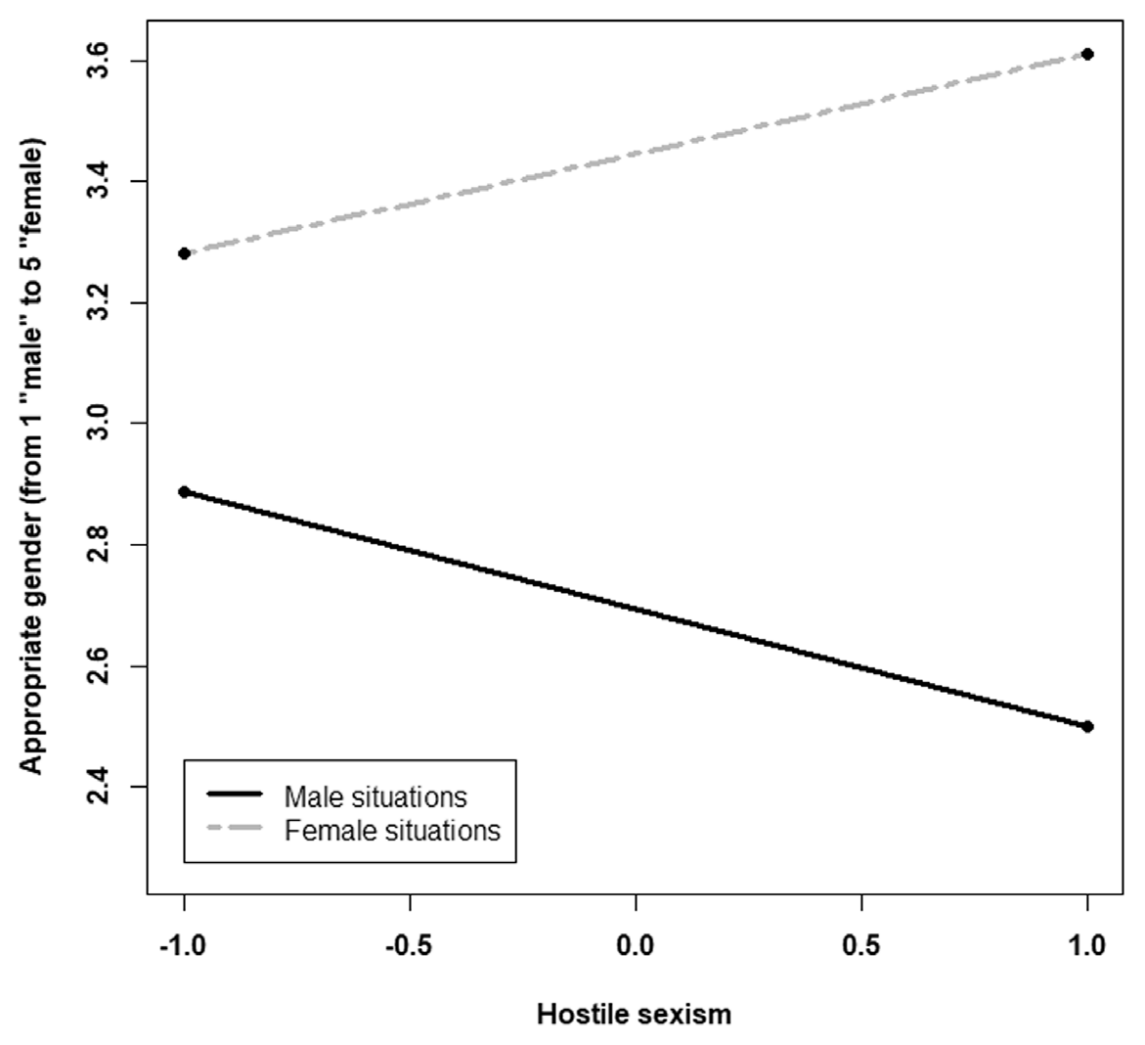
Conversational agents appropriate gender according to the gendered situations (represented by C1) and hostile sexism (centred). A lower value in the appropriate gender corresponds to a rather masculine gender, and a higher value corresponds to a rather feminine gender.

Moreover, the interaction between hostile sexism and C1 was significant, *t* = 3.82, *p* = .002, and the interaction with C2 was not, *p* = .90. The effect of the stereotypical situations increases with participants’ hostile sexism. The more sexist the participants are, the more they consider that the CAs’ gender should match the gendered situations (see Figure 2).

##### The Appropriate Level of Warmth

**Figure 3:**
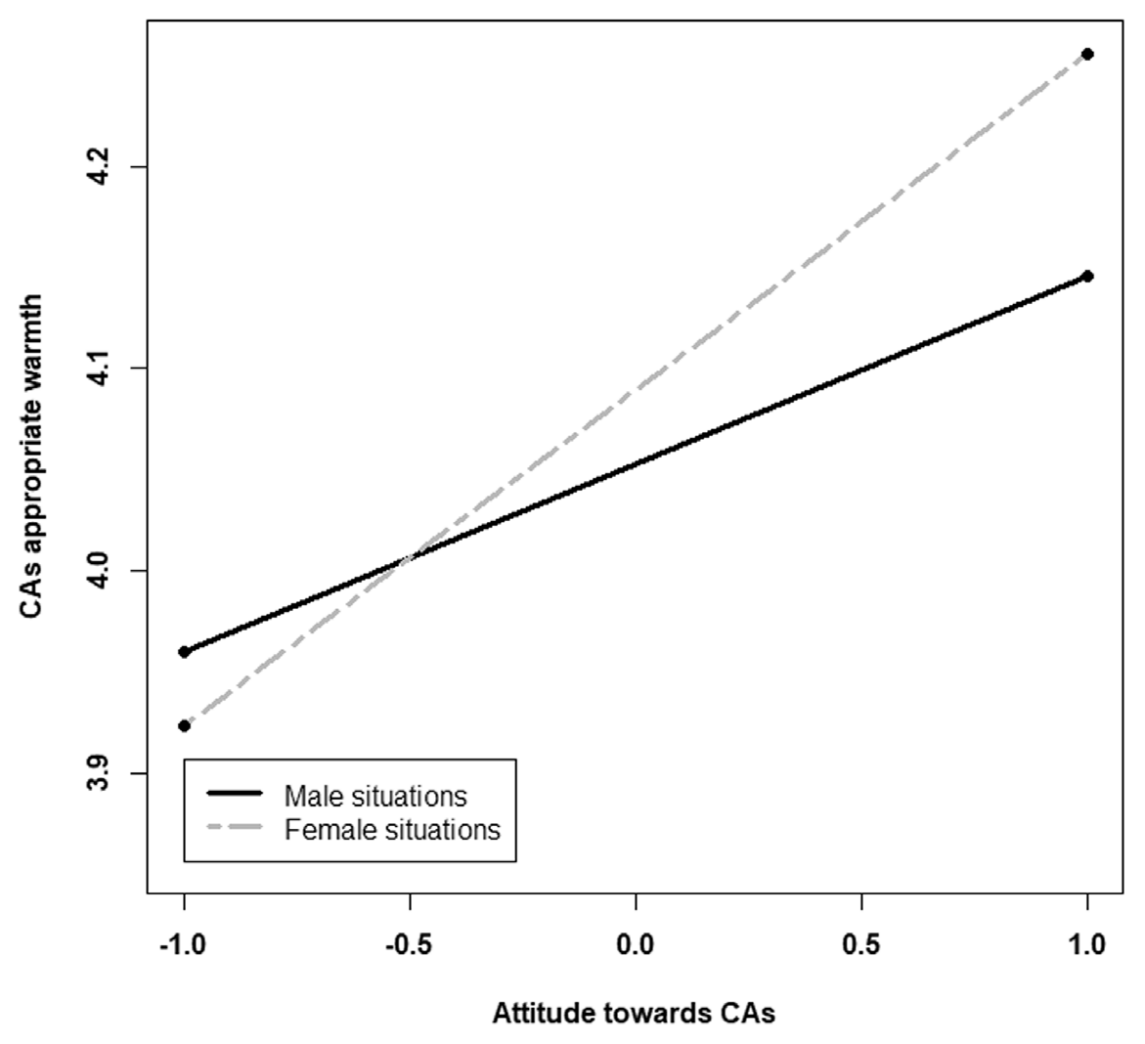
Appropriate level of warmth for the Conversational Agent according to the gendered situations (represented by C1) and attitude towards CAs (centred).

The suspicion level, the gender of the participants, and hostile sexism did not have a valuable input into the model and therefore were discarded. We did not find a significant effect of gendered situations on the appropriate level of warmth, C1: *t* < 1, *p* = .61, C2, t < 1, *p* = .36. We did not find a significant interaction with benevolent sexism. The interaction between the attitude towards CAs and C1 was significant, *t* = 2.91, *p* = .003, but not with C2, *p* = .66. Participants perceived warmth to be more appropriate in the female stereotypical situations than in the male stereotypical situations, when they have a more positive attitude toward CAs (Figure 3).

##### The Appropriate Level of Competence

Suspicion level, gender of participants, attitudes towards CAs, hostile sexism and benevolent sexism were found to have no valuable input in the model, so these variables were discarded. We found a significant effect of C1 on the appropriate level of competence, *t* = –3.03, *p* = .017, but not of C2, *p* = .51. The results showed a linear decrease in the appropriate level of competence when moving from stereotypically masculine situations (*M* = 4.62; *SD* = 0.34) to stereotypically female situations (*M* = 4.46; *SD* = 0.40).

#### Discussion of Study 1

The goal of this first study was to test the effect of stereotypically gendered situations on the expected features of a neutral conversational agent, according to hostile and benevolent sexism. The results partly support our hypothesis. The appropriate gender for a neutral CA was regarded as more female in stereotypically female situations and more male in stereotypically male situations, and this effect increased according to the level of hostile sexism. In addition, the competence traits were perceived more appropriate in male situations than in female situations. Interestingly, we did not find these effects in relation to warmth. Instead, the appropriate level of warmth was predicted by participants’ attitudes towards CAs differently in stereotypically male and female situations. Specifically, the more positive the participant’s attitude toward CAs is, the more the participants perceived warmth as appropriate in stereotypically female situations compared to stereotypically male situations. This effect was not expected and needs replication.

In this study we did not control the customer’s gender and used a within-subjects design: Participants were exposed to the nine situations. The within-subjects design may have increased the participants’ awareness of our hypotheses related to gender stereotypes. Furthermore, the customer’s gender being unspecified, the gendered situations may have influenced not only the CA’s perceptions but also the perceptions of the customer. Participants may have inferred that the customer is a woman in stereotypically female situations and a man in stereotypically male situations. This inference could have influenced participants through unexpected processes. Research shows that people prefer CAs that look like them and have a similar gender (ter Stal et al., 2020; Bailenson et al., 2008). Hence, men would prefer masculine CAs and women would prefer feminine CAs. However, to decrease social desirability, we asked participants to take the perspective of an average customer (not their own perspective), and interestingly we found no effect of the participants’ gender. However, in order to meet the requirements of the task (i.e., rate the appropriate level of traits to increase the customer satisfaction), participants could have answered based on the two uncontrolled inferences that the customer is a woman (a man) in female (male) situations and based on their gut feeling that a female (male) customer would prefer a female (male) agent to match the customer gender. Since we want to ascertain that the gendered situations influence the perception of the agent and answers are not related to the customer gender, we decided to manipulate the customer gender in Study 2.

Thus, we conducted a second study, with a larger sample, in which we controlled the gender of the customer and used a between-subjects design to minimize any awareness about our hypotheses, by limiting the number of situations presented.

### Study 2

In this study, we aimed to replicate the results of Study 1 and to overcome its limitations. We presented only one gendered situation type (male, neutral, or female) using a between-subjects design, and we presented the customer as either a man or a woman. We formulate the following hypothesis:

H2a: Participants would consider the male gender more appropriate for the CA in stereotypically male situations and the female gender more appropriate in stereotypically female situations, regardless of the customer’s gender.

H2b: Participants would deem warmth features more appropriate for the CA in stereotypically female situations and competence features more appropriate in stereotypically male situations, regardless of the customer gender.

H2c: We expected the effect of the stereotypically male and female situations to be moderated by sexist attitudes such that the more sexist (hostile or benevolent) the participant is, the more they would rely on gender stereotypes in their evaluation of CAs, regardless of the customer gender.

#### Method

##### Participants

Three hundred and eighteen persons participated in this online study (the result of the power analysis was *N* = 245, which was carried out to detect main and interaction effects with an effect size of *f* = .20, a power of .80, and using a between-subjects design). French-speaking participants were recruited on the Prolific platform (participants with 95% of positive rates were included) and received £0.84 for their participation, similar to the first study. Forty-seven participants were discarded (based on the time taken to fill out the study and the distraction level). The final sample included 271 participants (*M*_age_ = 29.84, *SD* = 10.44; 113 women and 158 men). We performed a sensitivity analysis to indicate what effect size was detectable with the final sample (setting an error alpha rate of .05, a power of .80, 271 participants and 6 groups). The analysis indicated that with this design, the minimum effect we could detect would be a *f* = .18 (*d* = .36).

##### Material and Procedure

Similar to Study 1, Study 2 was presented as two supposedly separate studies. Participants completed the short version of the ambivalent sexism questionnaire (Rollero et al., 2014) and answered questions about the CA presented in one situation. Unlike Study 1, we fixed the gender of the customer, and the participants were presented with only one situation randomly selected among the nine different situations (3 male, 3 neutral, and 3 female situations). The instructions specified the customer’s gender (e.g., ‘this customer is a woman’ or ‘this customer is a man’) in each situation. We used the same questionnaire about the CA’s appropriate features as in Study 1. An item was added to the post-experimental questionnaire to check if participants correctly recalled the gender of the customer.

#### Results

##### Data Preparation

The dependent variables were the same as in the first study. We obtained the perceived appropriate gender of the CA (1 = male to 5 = female), an appropriate level of warmth index (mean evaluation of warmth traits from 1 = not at all to 5 = very much), and an appropriate level of competence index (mean evaluation of competence traits from 1 = not at all to 5 = very much).

An analysis of covariance^8^ was run to test our hypothesis. We used four independent variables in a between-subjects model. The first independent variable (IV) was the stereotypically gendered situation, with three categories (male, neutral, female). To decompose omnibus effects, as in Study 1, we tested a linear effect with a contrast C1 (female = +1; neutral = 0; male = –1) and a residual contrast C2 (female = –1; neutral = +2; male = –1). The second IV was the customer’s gender, with two categories (male or female). Benevolent sexism (centred) and hostile sexism (centred) were the third and fourth IVs entered in the model as continuous variables. All main effects, one-way, two-way, and three-way interactions were tested (complete model). Control variables (participants’ gender, suspicion level, and attitudes towards CAs) and their interaction with the gendered situation IV were added and their impact was tested for each measure (as suggested by the comparison model approach of Judd et al., 2017). The results did not show a significant impact of the control variables on the explained variance and interactions with the IVs of interest, and they were discarded from the analysis. Similarly to the first study, all measures showed acceptable internal constancy (see Table 2). Two outliers (one for the appropriate gender and one for the appropriate level of warmth) were detected with the cook’s distance and discarded from the analysis (see Judd et al., 2017). The analysis was performed on 270 participants for the appropriate gender and appropriate warmth and 271 for the appropriate competence.

**Table 2:** Means (*SD*) and Cronbach’s alpha of variables included in the model (study 2).


	MEAN (*SD*)	CRONBACH’S ALPHA

Hostile sexism	2.32 (1.14)	.90

Benevolent sexism	2.84 (1.12)	.83

Appropriate competence	4.47 (0.47)	.69

Appropriate warmth	4.00 (0.63)	.70

Attitudes toward CAs	4.46 (1.16)	.88


##### The Conversation Agent Appropriate Gender

**Figure 4:**
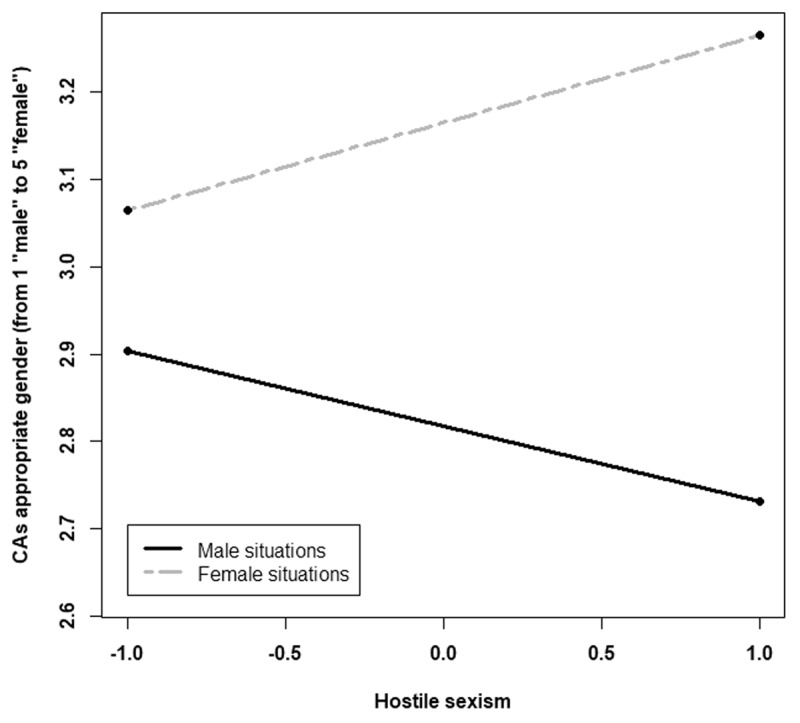
Conversational agent’s appropriate gender according to the gendered situations (represented by C1) and hostile sexism (centred). A lower value in the appropriate gender corresponds to a rather masculine gender, and a higher value corresponds to a rather feminine gender.

The analysis indicated a significant effect of the stereotypically-gendered situation on the appropriate gender of the CA, *F*(2, 246) = 10.06, *p* < .001, η^2^_p_= .0.07. As we expected, the decomposition of this effect indicated a significant effect of C1, *F*(1, 246) = 17.44, *p* < .001, η^2^_p_= .06, *B* = 0.17, 95% CI [0.09, 0.25], but not of C2, *p* = .15. The appropriate gender increased linearly toward femininity when passing from stereotypically masculine situations (*M* = 2.84; *SD* = 0.54) to stereotypically female situations (*M* = 3.23; *SD* = 0.47). Moreover, we observed a significant interaction between the stereotypically gendered situation and hostile sexism, *F*(2, 246) = 3.27, *p* = .039, η^2^_p_= .02. Specifically, the interaction between hostile sexism and C1 was significant, *F*(1, 246) = 5.22, *p* = .023, η^2^_p_ = .02, *B* = 0.09, 95% CI [0.012, 0.17], but not the interaction with C2, *p* = .23. As predicted, the higher the participant’s hostile sexism score of the participant, the more the gendered situation influenced their evaluation of the appropriate CA gender (Figure 4). The gender of the customer had no main effect, *F*(1, 246) = 0.76, *p* = .38, no significant interaction effect with the situation, *F*(1, 246) = .40, *p* = .66, nor with the ambivalent sexism (*Fs* < 1).

##### The Appropriate Level of Warmth

We did not observe the main effect of stereotypically gendered situations, *F*(2, 246) = 1.98, *p* = .14, and no effect of the customer gender on the appropriate level of warmth, *F*(1, 246) = 0.12, *p* = .72. Moreover, hostile sexism and benevolent sexism did not interact with stereotypical situations or with the customer gender.

##### The Appropriate Level of Competence

A significant effect of stereotypically male and female situations was found, *F*(2, 247) = 7.21, *p* < .001, η^2^_p_ = .05. The decomposition of this effect indicated an effect of C1, *F*(1, 247) = 9.46, *p* < .01, η^2^_p_ = .037, *B* = –0.10, 95% CI [–0.17, –0.03], and of C2, *F*(1, 247) = 5.71, *p* = .017, η^2^_p_ = .02, *B* = –0.05, 95% CI [–0.09, –0.009]. This effect was not linear (M_male_= 4.62; *SD*_male_ = 0.36, M_neutral_= 4.36; *SD*_neutral_ = 0.45, M_female_ = 4.46; *SD*_female_ = 0.40). Participants perceived that competence traits were more appropriate in male gendered situations than in the female ones, but also more than in the neutral ones. We also observed a significant main effect of hostile sexism on the appropriate level of competence, *F*(1, 247) = 7.94, *p* < .01, *B* = –0.08, 95% CI [–0.14, –0.02]. The higher the sexism of the participants, the greater the attribution of competence to the CAs. Hostile and benevolent sexism and customer gender did not significantly interact with stereotypically male and female situations, nor together.

### Discussion of Study 2

The goal of this second study was to replicate the results of the first study and control the effect of the customer’s gender on the CA’s appropriate gender, warmth and competence. We manipulated the customer gender between-subjects (the customer was either a man or a woman). Given the work on CA preference, gender, and gender stereotypes (ter Stal et al., 2020; McDonnell & Baxter, 2019; Brahnam & De Angeli, 2012), we reasoned that the gender of the customer may influence the perception of appropriate features for the CA in such a way that manly features in the CA could be seen as more appropriate for male than female customers (and vice versa). While a majority of participants correctly recalled the customer gender (69% of participants did so), we did not find any main or interaction effect of the customer gender on the appropriate gender, appropriate level of warmth, or appropriate level of competence of the CA. Our results are not related to the customer gender or to any expected match between the customer gender and the conversation agent gendered features.

We used a between-subjects design in which participants were presented with one kind of situation (either a stereotypically male, or a stereotypically female, or a gender-neutral situation). The moderation effects of hostile and benevolent sexism were tested in the same way as in the first study. The results replicated the influence of the stereotypically gendered situations on the CA’s appropriate gender observed in the first study, and its interaction with hostile sexism. We partially replicate the effect of the gendered situations on the appropriate level of competence such that competence was perceived as more appropriate in the male situations than in the female situations. However, in Study 2, we also found that competence was perceived to be more appropriate in both gendered situations than in neutral situations. We did not expect that pattern. In addition, hostile sexism predicted the appropriate level of competence such that the more sexist the participants, the more competence they expect from the CA. However, the level of (hostile) sexism did not interact with the gendered situation to predict the appropriate level of competence. Finally, we did not replicate the interaction effect of attitudes toward CAs and gendered situations on the appropriate level of warmth. The appropriate level of warmth did not vary as a matter of the gendered situations nor the level of sexism.

## General Discussion

The purpose of this paper was to empirically test the effect of gendered situations on the perceived appropriate features (gender, warmth, and competence) of neutral CAs in a customer service context, according to the participant’s level of hostile and benevolent sexism. Several stereotypically male and female situations were presented to participants in our two studies. Their task was to rate the appropriate characteristics for the conversational agent in each situation. Some of the results were in line with our hypotheses.

The participants judged that female CAs were more appropriate in stereotypically female situations and male CAs were more appropriate in stereotypically male situations, even when the CA has itself no gender features. Hostile sexism moderated some of the effects such that the more hostile sexist the participants are, the more stereotypical their perceptions were. However, benevolent sexism did not predict nor moderate any effect.

In both studies, competence traits were rated as more appropriate in stereotypically male situations than in stereotypically female situations. This effect was not significantly moderated by the level of sexism in any of the studies. In Study 2, we unexpectedly found that competence was perceived as more appropriate in stereotypically male and female situations than in neutral situations. This effect did not appear in Study 1 in which all scenarios were rated in a within-subject design. This effect may be related to the change of design from within-subjects to between-subjects designs. Also, hostile sexism had a positive main effect on the appropriate competence level. Replication is needed.

We did not find any consistent effect of gendered situations on the appropriate level of warmth traits. In Study 1, it interacted with the attitude toward the CAs to predict the appropriate level of warmth but in Study 2, it did not influence the dependent variable at all. Also, we did not find any impact of the participants’ sexism level on the appropriate level of warmth. Warmth does not seem as relevant for CAs as competence or gender and is not influenced consistently by the situation.

These results are in line with previous studies showing that gender stereotypes apply to robots (e.g., Bernotat et al., 2021). We extend these results by showing that gender stereotypes apply to conversation agents with no social features. We found that the mere kind of service required from the CA is enough to trigger stereotyping. For example, searching for banking advice triggered male stereotyping of the CA while searching for a beauty device triggered female stereotyping of the neutral CA. Here, we highlight that gender stereotypes creep into the smallest of gaps and that gender stereotypes matter for the conception of CAs. More precisely, we show that digital customer service situations convey gendered expectations that are usually observed in social interactions between humans, and that the specific digital situation affects the CA’s expected features (i.e., their congruent gender and traits). In agreement with the CASA theory research (e.g., Feine et al., 2019), we show that the gender rules apply in a digital customer service context with nonhuman CAs. Also, we show that hostile sexism but not benevolent sexism moderates some expectations of features in CAs.

Why did hostile but not benevolent sexism moderate our effects? The first explanation could lie with the Online Disinhibition Effect (Suler, 2004), stating that people experience diminished constraints because online anonymity decreases inhibition and increases self-disclosures (Hollenbaugh & Everett, 2013; Stuart & Scott, 2021). In the same way, Brahnam and De Angeli showed that people could be abusive toward virtual agents, mostly with ‘female’ CAs, explaining this effect as agent-induced disinhibition (Brahnam & De Angeli, 2012; Brahnam, 2006; De Angeli & Brahnam, 2008). Situations in our studies are not hostile, but online stereotypical situations diminish constraints, foster disinhibition, and can prime hostile sexism attitudes, explaining the moderation effect of hostile sexism. Although hostile and benevolent sexism are interrelated (Glick & Fiske, 2001), hostile sexism could have taken over benevolent sexism in our studies.

Interestingly, Bernotat et al. (2021) suggested that benevolent sexism was more socially appropriate than hostile sexism to justify their findings on the relationship between the judgment of a robot and benevolent sexism. However, they used visual representations of robots that could have activated benevolent sexism rather than hostile sexism. In our study, we only define what a (neutral) conversation agent is and that seems to have activated hostile sexism instead of benevolent sexism. Another explanation is that the task in our studies was perhaps less prone to social desirability. In fact, participants rated CAs’ features for ‘average internet users’ and not directly for themselves, an approach supposed to decrease social desirability (e.g., Fiske et al., 2002). Hence, they may have felt allowed to express their hostile attitude rather than their more controlled and polished benevolent attitude. The last explanation could be also linked to the (lack of) suspicion of participants regarding the link between the studies. Suspicion could have led participants to control their responses and resist the influence of independent variables (as some kind of reactance effect). Here, suspicion did not moderate our results or the effects of the IVs. It appears that participants were not controlling their answers and hence let their hostile attitude do its job. We had no specific hypothesis toward hostile or benevolent attitudes, both being the two sides of the same medal. Future research should more thoroughly test when hostile vs. benevolent sexist attitudes predict people’s perceptions of robots and conversational agents.

Our studies differs from previous research on several points. Participants are usually asked to choose tasks to which robots or virtual agents could be suited (e.g., Bernotat et al., 2021; Eyssel & Hegel, 2012; Forlizzi et al., 2007). Our studies focused instead on the features users in real life might want in a customer service context. Moreover, we designed stereotypically male and female situations and used a gender-undefined CA, rather than using gendered visual representations of the CAs. We believe that this situation design increases the external validity of our results because the required services (e.g., banking advice vs. bank opening hours) are all plausible for every human being, male or female. These situations may happen outside the lab for all of us. Here, gender stereotyping was not triggered by specific visual representations of the CA as used in previous work (e.g., Brahnam & De Angeli, 2012; Forlizzi et al., 2007; McDonnell & Baxter, 2019).

This paper raises the inevitable question of ethics in the development of robots and applications. Our results indicated that the cognitive biases of human interactions also apply to interactions with CAs. Previous papers have debated whether to rely on gender stereotypes when developing robots (and thus CAs) to improve the user experience (e.g., in terms of credibility and trustworthiness), or to develop neutral gendered robots (and thus CAs) to prevent cognitive biases (e.g., Eyssel & Hegel, 2012). Forlizzi and their collaborators showed that people prefer CAs corresponding to their stereotypes (Forlizzi et al., 2007). To manage both customer satisfaction and mitigation of gender stereotypes, one option may be to provide some choices regarding CA features to the users, while avoiding stereotypical features or features that may be associated with discrimination in humans. Another solution could be to foster human characteristics without gender as an androgynous face or voice (e.g., Nag & Yalçın, 2020) to avoid repeating gender stereotypes. Indeed, it was shown that a gendered conversational agent is the target of more sexist and harrassive talk than a gender-neutral agent (e.g., Brahnam & De Angeli, 2012; Brahnam, 2006; De Angeli & Brahnam, 2008). Gender-neutral agents are less verbally-abused than gendered ones and thus gender-neutral agents do not fuel so much into stereotypes, prejudice and discrimination. They would contribute less to any normalization, banalisation, or justification of sexism in real life (see, for instance, Fox et al., 2015). These studies have shown that gender-undefined CAs may still be the target of gender stereotyping depending on the users’ level of hostile sexism. Gender stereotyping is so widespread that the type of required service is enough to trigger gender expectations about the agent. Given that gender-neutral agents trigger less sexual and harassive talks than female agents (e.g., Brahnam & De Angeli, 2012) and that our work shows that the mere type of customer service triggers gendered expectations from sexist users, we believe developers should limit to the minimum any feature that may reinforce gender stereotyping.

### Limits and Future Studies

We did not check the participants’ mental representations of the conversation agent. Other studies have signalled a visual representation to participants (e.g., Brahnam & De Angeli, 2012; McDonnell & Baxter, 2019), allowing them to control the mental representation of CAs. Future studies could address this limit with more ecological situations. For example, a proper interaction with a CA could be designed on a professionally-designed customer service website to test participants’ inferences, wishes, and satisfaction.

There are a wide variety of social cues, which can be classified into several categories such as verbal, visual, auditory, or invisible (Feine et al., 2019). These characteristics affect the perception of CAs, such as social presence, trust, satisfaction, and credibility (Chung et al., 2020; Araujo, 2018; de Visser et al., 2016; Verhagen et al., 2014; Demeure et al., 2011). We can suppose that stereotype activation is stronger when social cues are salient than in situations without social cue. For example, we can imagine that a female voice-based CA is a stronger female social cue than a text-based CA with a female name. It will be pertinent for future studies to compare these types of CAs according to these social cues.

Another point to note in our studies is that the participants had to indicate the appropriate characteristics of CAs. The research shows that gender stereotype activation, notably in the workplace, is higher when people transgress their expected gender role (e.g., Koch et al., 2015; Prentice & Carranza, 2002). For example, the more inconsistency there is between a role in the workplace and the expected gender features, the lower performance is expected from the (human) agent (Eagly & Karau, 2002; Heilman & Parks-Stamm, 2007; Stamarski & Son Hing, 2015). In future studies, it would be interesting to test situations with varying consistency between the CA’s role (e.g., expected competence vs. warmth) and the CA’s features (e.g., masculine vs. feminine). Explicit and implicit attitudes towards this CA can be tested in relation to varying role-features’ consistency.

Finally, another limitation of the present studies lay in the direct questionnaire that measures sexism. In the future, it would be appropriate to measure the participants’ level of sexism using implicit or indirect measures (e.g., Oliveira Laux et al., 2015) to limit the suspicion of the participants and subsequently the phenomenon of social desirability. Although our study focused only on benevolent and hostile sexism, it would be interesting to consider other moderators to understand under which conditions gender stereotypes are applied to CAs. For example, one moderator could be the acceptance of new technology, which has been related to the use of CAs (e.g., Zarouali et al., 2018; Rese et al., 2020). Indeed, several studies showed that the acceptance of new technologies depended on their conformity with social norms (see Legris et al., 2003). The more normative the technology, the more acceptance it will get. From this perspective, we can assume that participants who adhere to gender norms would accept new technologies more when the technologies convey gender stereotypes that are consistent with their sexist attitudes. In this perspective, future work could test the effect of gender stereotypes on the use and acceptance of new technologies, such as CAs, depending on whether these new technologies have social aspects that are consistent with gender stereotypes or not.

To conclude, we have shown in these two studies that gender stereotypes apply to the perception of CAs in a similar way to our projection of the social roles observed in reality and as has been highlighted in social psychology. We have also shown for the first time that hostile sexism moderates gender stereotypes in the perception of CAs. Although. in 2012 it was suggested that we would have robot assistants in the future (Eyssel & Hegel, 2012), in 2022 we do not yet have personal robot assistants at home. However, gender-related stereotypes in computers seem not to be a thing of the past. Researchers and professionals should work closely together to minimize negative gender biases when developing conversation agents.

## Data Availability

Data are available here: https://osf.io/ycqrx/.

## Footnotes

https://venturebeat.com/2018/05/01/facebook-messenger-passes-300000-bots/.

https://theconversation.com/theres-a-reason-siri-alexa-and-ai-are-imagined-as-female-sexism-96430 – https://thechatbot.net/chatbot-male-female-gender-neutral/.

We controlled the time to complete the study and whether participants were distracted during the study with a single item (‘*I was distracted during the study*’ from 1 = strongly disagree to 7 = strongly agree; e.g., Meade & Craig, 2012). Participants completed the questionnaire in an average of 14 minutes. We excluded participants who took more than 30 minutes (M+2SD), as well as participants who declared that they were distracted during the study (answers above 3 on the 1–7 points scale).

The study was performed in French, and we used the neutral term ‘un(e) client(e)’, which did not specify whether the customer was male or female.

The situations were pre-tested on 20 participants to make sure that they matched the expected gender norms. Pre-test participants were asked: *To what extent would the CA have masculine or feminine characteristics?* (scale of 1 = male to 5 = female) (In french: *Dans quelle mesure l’agent conversationnel aurait des caractéristiques masculines ou féminines*?). Situations designed to be masculine led the participant to expect more masculine CAs (*M* = 2.48) than the situations designed to be feminine (*M* = 3.7). The situations designed to be neutral fell in between (*M* = 3).

In our results, we report fixed effects but not random effects, because the latter are not relevant to the purpose of this paper.

We checked normality for each model and compared the results with and without a Box-Cox transformation. The results were very similar with and without Box-Cox transformations so we decided to present the results on untransformed DVs. However, for the competence DV, the Box-Cox transformation did remove a significant interaction between hostile sexism and gendered situations that appeared with the untransformed DV. For this DV, we present the results after Box-Cox transformation. The results reported here are the least favourable in regards of our hypotheses.

For this second study, we did not use a Mixed Models Analysis, because participants were not exposed to the three conditions of the stereotypically gendered situations (female, neutral and male) but only to one condition.

## Appendix

The male gender norm situations were as follows:

**Table:**

A client wishes to open a bank account to save their money and make investments. The client requests a conversation agent on the bank’s website to find out more about the type of financial services available to save and invest money.

A customer has technical questions about the operation of a handiwork product when browsing through the section of a commercial website. The customer requests a conversation agent on the website to ask technical questions about the handiwork product.

In order to purchase a computer product, a customer wishes to receive information regarding the payment in installments offered by a commercial website. The customer requests a conversation agent on the website to obtain information about this financial service.

The female gender norm situations were as follows:

**Table:**

A customer wishes to know the opening hours of a banking establishment. The customer requests a conversation agent on the bank’s website to obtain information about the opening hours.

A customer does not find a desired beauty product while browsing on a commercial website. The customer requests a conversation agent on the website to find the beauty product.

A hairdressing appliance purchased by a customer on a commercial website has broken down. The product is still under warranty. The customer requests a conversation agent on the website to find out how to use the guarantee attached to the hairdressing appliance.

The neutral gender norm situations were as follows:

**Table:**

A customer’s new bank card has not yet arrived at their home. The customer requests a chat agent on the bank’s website in order to get information about the sending of their new card.

A customer wishes to know when a product indicated as ‘out-of-stock’ on a commercial website will be available again. The customer requests a conversation agent on the website to obtain information on the product’s restocking.

A customer wishes to have information on the type of delivery offered by a commercial website. The customer requests a conversation agent on the website to get information about the delivery service(s) offered.
